# Total, Free, and Added Sugar Consumption and Adherence to Guidelines: The Dutch National Food Consumption Survey 2007–2010

**DOI:** 10.3390/nu8020070

**Published:** 2016-01-28

**Authors:** Diewertje Sluik, Linde van Lee, Anouk I. Engelen, Edith J. M. Feskens

**Affiliations:** Division of Human Nutrition, Wageningen University, Wageningen 6700 AA, The Netherlands; lindevanlee@hotmail.com (L.L.); Anouk.Engelen@wur.nl (A.I.E.); edith.feskens@wur.nl (E.J.M.F.)

**Keywords:** sugars, added sugars, free sugars, survey, guidelines, diet quality

## Abstract

A high sugar intake is a subject of scientific debate due to the suggested health implications and recent free sugar recommendations by the WHO. The objective was to complete a food composition table for added and free sugars, to estimate the intake of total sugars, free sugars, and added sugars, adherence to sugar guidelines and overall diet quality in Dutch children and adults. In all, 3817 men and women (7–69 years) from the Dutch National Food Consumption Survey 2007–2010 were studied. Added and free sugar content of products was assigned by food composition tables and using labelling and product information. Diet was assessed with two 24-h recalls. Diet quality was studied in adults with the Dutch Healthy Diet-index. Total sugar intake was 22% Total Energy (%TE), free sugars intake 14 %TE, and added sugar intake 12 %TE. Sugar consumption was higher in children than adults. Main food sources of sugars were sweets and candy, non-alcoholic beverages, dairy, and cake and cookies. Prevalence free sugar intake <10 %TE was 5% in boys and girls (7–18 years), 29% in women, and 33% in men. Overall diet quality was similar comparing adults adherent and non-adherent to the sugar guidelines, although adherent adults had a higher intake of dietary fiber and vegetables. Adherence to the WHO free sugar guidelines of <5 %TE and <10 %TE was generally low in the Netherlands, particularly in children. Adherence to the added and free sugar guidelines was not strongly associated with higher diet quality in adults.

## 1. Introduction

A high intake of sugars is subject of scientific debate due to its suggested health implications, mainly on obesity risk. Up until now, most studies have focused on the consumption of sugar-sweetened beverage (SSB) consumption and free or added sugars [1,2]. A higher intake of free sugars and SSB has been associated with a higher risk of dental caries [3,4,5] and obesity [6]. In their review of 30 trials and 38 cohort studies, Te Morenga, Mallard and Mann found that a higher *ad libitum* intake of free sugars and SSB was associated with weight gain [6]. Although this association was driven by energy intake [6], results from randomized controlled trials suggest that there is inadequate energy compensation for energy delivered from sugars [2]. As a result, increasing sugar intake increases energy intake in free-living individuals consuming *ad libitum* diets [2,7]. However, it is not clear whether this is restricted to liquid calories from SSB or to sugars from the total diet [8]. Moreover, the British Scientific Advisory Committee on Nutrition (SACN) conclude there is limited evidence for an effect of total sugars intake on cardio-metabolic outcomes and chronic diseases, mainly due to a paucity of studies [2]. However, there is consistent evidence that a higher SSB intake is associated with an increased risk of diabetes mellitus type 2 [9].

The terms total sugar, added sugars, and free sugars are often being used interchangeably in literature and recommendations. Total mono- and disaccharides or total sugars comprise intrinsic sugars, lactose in milk, and free sugars, but no exact uniform definition of added and free sugars exist. In general, added sugars comprise all sugars that are added during food manufacturing and preparation. Free sugars include added sugars as well as sugars that are naturally present in honey, syrup, fruit juices, and fruit concentrates [1].

Because they are not chemically different from natural occurring sugars, most food composition tables do not include information on the added and free sugars content of foods. Hence, researchers often have to rely on incomplete food composition tables or supply data. As a result, few countries have reported on intakes of added and free sugars intake. Recently, Newens and Walton have reviewed current intakes of dietary sugars from national representative dietary surveys across the world. Added sugar intake in adults have only been reported in nine countries and ranged from 7.2% Total Energy (%TE) in Brazilians aged 10 years and older in 2008–2009 to 16.3 %TE in U.S. adults aged 18–34 years in 2007–2008 [10].

Several guidelines with respect to intake of added and free sugars have been published. Most recently, the SACN advised that the population average intake of free sugars in the United Kingdom should not exceed 5 %TE free sugars [2]. In addition, in 2015, the World Health Organization (WHO) revised their guidelines for sugar intake to a strong recommendation for a consumption <10 (%TE and a conditional recommendation for a consumption <5 %TE [1]. The Health Council of the Netherlands has not set quantitative reference intakes, but a level of 20 %TE added sugars is mentioned, from where higher intakes may be associated with micronutrient dilution of the diet [11]. Furthermore, the Nordic Nutrition Recommendations (NNR) 2012 advise an intake <10 %TE added sugars to ensure an adequate intake of micronutrients and dietary fiber, especially in children and people with a low energy intake [12].

To gain more insight in the consumption of sugars, it was aimed to estimate the intake of total sugars, added and free sugars and their main food sources in children and adults. To this purpose, a food composition table for added and free sugars will be completed and linked to individual intake data from a representative subsample of the Dutch population. Moreover, the adherence to the recommendations of the SACN, WHO, Dutch Health Council, and the NNR was assessed as well as overall diet quality of those adherent to the guidelines.

## 2. Materials and Methods

### 2.1. Study Design and Population

The Dutch National Food Consumption Survey (DNFCS) 2007–2010 was conducted among 3819 Dutch men and women aged 7–69 years old [13]. Participants were recruited from consumer panels of a marketing research institute. Exclusion criteria were involvement in a food consumption survey during the previous four years, being pregnant or lactating, living in an institution, or an inadequate level of the Dutch language. A total of 5502 persons were invited, of which 3819 (69%) responded. The survey population was representative for the Dutch population with regard to age and sex within each age group, region, degree of urbanization and educational level. Data were collected from March 2007 to April 2010. Age-specific general questionnaires assessed socio-demographic information; educational attainment and income level were assessed from age 12. Diet was assessed by 24-h dietary recalls. Two persons who reported no dietary intake during one recall were excluded, resulting in an analytical sample of 3817 participants. The study was conducted according to the guidelines laid down in the Declaration of Helsinki. Written informed consent was obtained from all subjects.

### 2.2. Dietary Assessment

Trained dieticians conducted two non-consecutive 24-h recalls by telephone using the computer directed interview program EPIC-SOFT. EPIC-SOFT follows standardized steps for 24-h recalls and has been described in detail elsewhere [14,15]. Children aged 7–15 years were interviewed face to face during a home visit where at least one of the child’s parents or caregivers was present. Participants aged 16 and older were interviewed by telephone. The interviews comprised the following steps: (1) General participant information, including special information on the day of the 24-h recall; (2) A quick list for each food consumption moment; (3) Description and quantification of foods reported in the quick list including preparation method and portion size; and (4) Any further information including the use of dietary supplements. Each person was interviewed twice with an interval of 2–6 weeks. The recalls were spread over all days of the week and seasons. Food consumption on Sunday to Friday was recalled the next day; consumption on a Saturday was recalled on the following Monday. All surveys were linked to the Dutch National Food Composition Table 2011 [16]. Average macro- and micronutrient and food intake over the two days was calculated and foods were organized into 17 food groups and 85 subgroups according to the EPIC-SOFT classification; only food groups that contributed to sugar intake are presented.

### 2.3. Dutch Healthy Diet-Index

Diet quality was estimated using the Dutch Healthy Diet-index (DHD-index) score, an *a priori* diet score [17,18]. The DHD-index comprises ten components representing the 2006 Dutch Guidelines for a Healthy Diet as proposed by the Dutch Health Council. Each component score could range between 0 and 10 points. Ten points were allotted for complete adherence to the guidelines, resulting in a total score between 0 and 100 points. The ten components include adherence to the recommendations on physical activity, vegetables, fruit, dietary fiber, fish, saturated fatty acids, trans fatty acids, consumption occasions with foods and beverages that contain easily fermentable sugars and drinks that are high in food acids, with respect to dental health, sodium, and alcohol. Physical activity was assessed by the Short Questionnaire to Assess Health enhancing physical activity (SQUASH) [19].

### 2.4. Food Composition Table for Total, Added and Free Sugars

The Dutch food composition table of 2011 was used to assign total sugars [16]. This table includes 2556 food items, among which unbranded and branded items. Added sugars were defined as all sugars that are added during food manufacturing and preparation. Raw, white, and brown sugar, honey, and syrups are assumed to be added during food preparation and are thus considered as added sugars. Added sugars did not include naturally occurring sugars found in unprocessed products (fruit, vegetables, legumes, potatoes, fish, meat, poultry, and eggs), juices, fruit concentrates, bread, and lactose in dairy. Free sugars were defined according to the definition of the WHO as “all sugars that are added during food manufacturing and preparation as well as sugars that are naturally present in honey, syrups, fruit juices and fruit concentrates” [1].

Added sugar content of 2556 food items was systematically assigned by product group, largely based upon the criteria for the food labelling system on front package of the International Choices Programme [20]. Supplementary Table S1 gives an overview of the decisions made and the number of food items assigned by product group. Next to the 2011 Dutch food composition table, the Danish food composition table [21] (*n* = 30), standard recipes (*n* = 93), information on packaging (*n* = 89), and information from manufacturers websites (*n* = 59) were used as information sources. When information on added sugars was limited, the content was estimated as the amount of sugars minus the amount of natural occurring sugars estimated from a similar unsweetened food item (*n* = 375). Free sugars were calculated as added sugars, with the exception that all sugars naturally present in fruit juices and fruit concentrates were also included in the definition of free sugars. For 210 food items, free sugars content differed from added sugars content.

### 2.5. Statistical Analyses

SAS software (Version 9.3, SAS Institute Inc., Cary, NC, USA) was used for all analyses. Habitual daily sugars consumption was estimated using the Multiple Source Method (MSM) [22]. The MSM has been developed to predict habitual consumption based on a short-term measurement, such as the 24-h recall, and thereby reduces day-to-day variability. The percentage contribution of food groups was not estimated with MSM.

Age- and sex-specific intakes of total, added, and free sugars, %TE and %total carbohydrates or %total sugars were estimated. Furthermore, sugar consumption according to native country (the Netherlands or other) and net monthly income (three levels) was assessed. Difference in diet quality in adults adherent and non-adherent to the added and free sugars guidelines were assessed by independent *t*-test. All results were weighted for small deviances in socio-demographic characteristics, day of the week, and season of data collection, to obtain results that are representative for the Dutch population and for all days and all seasons. The weighing factors were formed in an iterative process using census data from 2008 as reference population [13].

## 3. Results

Median total sugars consumption was 115 g, contributing 21.5% of the total energy intake (Table 1). Less than half of the total carbohydrates (46.8%) were consumed as sugars. Median added sugar consumption was 64 g/day, comprising more than half (55%) of total sugars intake, and 12 %TE. Free sugar consumption was somewhat higher than added sugars consumption: median 74 g/day and 14 %TE. Boys and girls (7–18 years) had a higher sugar intake than adults (19–69 years). Men had a higher absolute sugar intake compared to women, but women consumed more energy from sugars compared to men. Sugar intake was comparable between native Dutch persons and persons born in other countries and only small differences in consumption were observed across income levels.

In both children and adults, the food groups “non-alcoholic beverages”, “sweets and candy”, “dairy”, and ”cake and cookies”, contributed most to intake of total, free, and added sugars (Table 2). In all age groups, “non-alcoholic beverages” contributed most to total and free sugars intake; contribution to total sugars intake ranged from 23.1% in women to 32.8% in boys and contribution to free sugars intake ranged from 34.4% in women to 40.3% in boys. “Sweets and candy”, in particular “sugar, honey, jams” and “chocolate” contributed most to added sugars intake: from 35.7% in boys to 36.9% in girls. As a single food item, “soft drinks” made the largest contribution to sugar intake: 21.1%–25.1% to total sugars intake in children and 11.3%–16.1% in adults and 25.5%–29.2% to free sugars intake in children and 17.4%–24.0% in adults.

Only one boy, two girls and 4% of the adult men and women adhered to the SACN recommendation and the conditional WHO- recommendation of consumption <5 %TE free sugars (Table 3). Percentage participants having a consumption <10 %TE of free sugars was 5% for boys and girls, 33% for men and 29% for women.

Overall diet quality, as assessed by the DHD-index, was similar between men and women adherent and non-adherent to the recommendations of the NNR, Health Council of the Netherlands, WHO, and SACN for added and free sugars (Table 4). Adherent adults scored higher to the components of vegetables and dietary fiber for all sugars guidelines, except for vegetable intake in men who consumed <5 %TE free sugars. Furthermore, adults who adhered to the <10 %TE and <20 %TE guidelines for added sugars scored higher for the components fruit and fish. On the other hand, adherent adults tended to score lower, *i.e.*, had higher intakes, for the components saturated fatty acids and alcohol for most guidelines. Overall, most statistical significant differences in diet quality were observed for the guidelines of <10 %TE and <20 %TE added sugar; for the free sugar recommendation <5 %TE, fewest differences in diet quality were observed.

**Table 1:** Weighted * habitual intakes of total sugars, added sugars, and free sugars in 3817 persons aged 7–69 years from the Dutch National Food Consumption Survey 2007–2010.

		Total Sugars			Added Sugars			Free Sugars		
	N	Median (P25-P75) ^1^	%TE, Mean (SD)	%Carbohydrates, Mean (SD)	Median (P25-P75)	%TE, Mean (SD)	%Sugars, Mean (SD)	Median (P25-P75)	%TE, Mean (SD)	%Sugars, Mean (SD)
All	3817	115 (90–147)	21.5 (6.1)	46.8 (9.2)	64 (40–93)	12.2 (5.8)	55.4 (16.4)	74 (49–106)	14.2 (6.2)	64.4 (15.5)
Men										
7–8 years	153	136 (116–166)	28.0 (3.4)	53.8 (4.4)	87 (72–110)	18.4 (3.0)	65.7 (5.9)	101 (81–124)	20.7 (3.3)	73.4 (5.4)
9–13 years	351	144 (124–173)	26.4 (3.3)	51.9 (4.4)	97 (74–122)	17.5 (3.3)	65.5 (7.2)	108 (86–134)	19.7 (3.5)	74.0 (6.5)
14–18 years	352	153 (124–188)	24.4 (3.7)	49.1 (5.3)	100 (76–132)	16.3 (3.7)	65.6 (8.7)	113 (87–145)	18.2 (3.9)	73.5 (7.8)
19–30 years	356	142 (105–179)	21.7 (6.4)	46.4 (10.1)	91 (60–120)	14.2 (6.4)	63.5 (14.7)	98 (70–137)	16.1 (6.6)	72.0 (13.1)
31–50 years	347	117 (90–158)	19.6 (8.2)	44.0 (13.6)	67 (40–100)	11.2 (7.8)	55.7 (25.3)	78 (49–114)	12.9 (8.1)	64.3 (24.1)
51–69 years	351	108 (81–135)	18.5 (6.6)	43.7 (11.0)	53 (36–76)	9.6 (5.2)	51.1 (18.5)	63 (41–84)	11.1 (5.7)	59.2 (16.8)
Women										
7–8 years	151	134 (115–152)	27.5 (2.9)	53.9 (3.9)	83 (70–103)	17.6 (2.9)	63.5 (6.8)	96 (79–114)	19.9 (2.9)	72.2 (6.2)
9–13 years	352	138 (116–162)	26.7 (3.4)	52.6 (4.7)	90 (71–111)	17.5 (3.2)	65.3 (6.8)	102 (83–123)	19.8 (3.3)	74.0 (5.8)
14–18 years	354	125 (102–149)	24.4 (3.5)	49.3 (5.3)	75 (55–99)	15.1 (3.4)	60.9 (8.6)	89 (68–112)	17.6 (3.6)	71.0 (7.3)
19–30 years	347	118 (91–146)	23.3 (6.0)	48.6 (8.9)	67 (44–96)	13.8 (5.9)	57.5 (16.2)	81 (52–109)	16.2 (6.5)	67.5 (15.2)
31–50 years	351	105 (83–130)	21.3 (7.9)	46.6 (11.8)	53 (35–76)	11.3 (7.2)	52.0 (21.3)	63 (45–87)	13.4 (7.8)	61.8 (20.3)
51–69 years	352	95 (75–118)	20.2 (6.1)	47.0 (9.9)	42 (28–57)	9.3 (5.1)	45.4 (18.1)	53 (37–69)	11.3 (5.7)	54.9 (18.2)
Native country										
The Netherlands	3701	116 (90–147)	21.4 (6.1)	46.8 (9.1)	64 (40–93)	12.2 (5.8)	55.4 (16.4)	74 (49–106)	14.2 (6.2)	64.4 (15.4)
Other	110	107 (87–144)	21.9 (6.4)	47.2 (10.2)	62 (35–88)	12.2 (6.6)	54.2 (18.9)	68 (40–97)	14.2 (7.0)	63.2 (18.7)
Net monthly household income										
Up to €1700	1197	117 (89–148)	21.7 (6.1)	47.1 (9.2)	64 (39–94)	12.5 (5.8)	55.9 (16.1)	76 (48–108)	14.5 (6.2)	65.0 (15.3)
€1700–€2500	1184	116 (91–148)	21.5 (5.9)	46.9 (8.8)	64 (40–97)	12.3 (5.7)	55.7 (15.8)	74 (50–109)	14.3 (6.1)	64.5 (14.9)
≥€2500	1436	114 (89–146)	21.2 (6.3)	46.5 (9.4)	63 (41–89)	11.9 (6.0)	54.7 (17.2)	73 (48–102)	13.9 (6.4)	63.8 (16.2)

* weighted for socio-demographic characteristics, day of the week, and season of data collection; ^1^ IQR: Inter-Quartile Range.

**Table 2:** Mean contribution (%) by food sources to intake of total sugars, added sugars, and free sugars in 3817 participants of the Dutch National Food Consumption Survey 2007–2010 according to age category and gender.

	**Boys, 7–18 Years**			**Girls, 7–18 Years**		
**Food Groups**	**Total Sugars (%)**	**Added Sugars (%)**	**Free Sugars (%)**	**Total Sugars (%)**	**Added Sugars (%)**	**Free Sugars (%)**
Potatoes, vegetables	1.5	0.1	0.1	1.5	0.1	0.1
Fruit, nuts, olives	6.3	0.9	0.8	7.9	1.0	0.9
Dairy	18.2	12.4	11.1	17.8	12.2	10.7
- Milk	5.8	0.0	0.0	5.6	0.0	0.0
- Dairy beverages	2.5	2.9	2.6	3.6	4.0	3.5
- Dairy desserts	3.2	3.4	3.0	2.3	2.7	2.4
- Yogurt	6.2	5.4	4.8	6.0	4.9	4.3
- Cottage cheese	0.3	0.5	0.5	0.2	0.4	0.3
- Cream, coffee milk	0.2	0.2	0.2	0.1	0.2	0.2
Bread	2.8	0.5	0.4	2.8	0.5	0.5
Breakfast cereals	0.8	1.0	0.9	1.0	1.3	1.2
Other grains and cereals	0.5	0.0	0.0	0.5	0.0	0.0
Sweets and candy	24.2	35.7	32.9	24.3	36.9	33.2
- Sugar, honey, jams	4.0	6.0	5.4	3.7	6.2	5.4
- Confectionary	5.3	8.4	7.5	5.3	8.5	7.4
- Chocolate	7.2	10.4	9.3	8.0	11.5	10.0
- Syrups	5.3	6.9	7.1	4.8	6.1	6.4
- Ice cream	2.4	4.0	3.6	2.5	4.6	4.0
Cake and cookies	9.3	10.0	9.0	10.3	11.5	10.1
Non-alcoholic beverages	32.8	34.2	40.3	30.2	31.1	38.7
- Fruit and vegetable juices	7.6	4.4	10.8	9.0	4.9	12.9
- Soft drinks	25.1	29.7	29.2	21.1	26.1	25.5
- Water	0.0	0.0	0.1	0.0	0.0	0.1
- Coffee/tea	0.1	0.1	0.2	0.1	0.1	0.2
Alcoholic beverages	0.2	0.3	0.3	0.6	0.9	0.9
Processed meat	0.4	0.4	0.3	0.4	0.3	0.3
Condiments and sauces	2.0	2.6	2.3	1.8	2.5	2.1
Soups	0.3	0.5	0.4	0.2	0.5	0.4
Miscellaneous	0.5	0.6	0.6	0.4	0.5	0.4
						
	**Men, 19–69 Years**			**Women, 19–69 Years**		
**Food Groups**	**Total Sugars (%)**	**Added Sugars (%)**	**Free Sugars (%)**	**Total Sugars (%)**	**Added Sugars (%)**	**Free Sugars (%)**
Potatoes, vegetables	2.6	0.2	0.2	3.3	0.4	0.2
Fruit, nuts, olives	9.5	0.8	0.7	13.2	1.0	0.8
Dairy	19.1	11.3	11.2	18.1	12.3	10.3
- Milk	7.1	0.0	0.0	6.1	0.0	0.0
- Dairy beverages	2.2	2.4	2.1	1.9	2.6	2.2
- Dairy desserts	3.4	4.0	3.5	2.8	4.2	3.5
- Yogurt	5.0	3.8	3.3	5.7	4.3	3.6
- Cottage cheese	0.5	0.8	0.7	0.6	0.8	0.7
- Cream, coffee milk	0.9	0.4	0.4	1.0	0.5	0.4
Bread	4.1	0.7	0.6	3.8	0.9	0.8
Breakfast cereals	0.9	0.8	0.7	1.2	1.2	1.0
Other grains and cereals	0.5	0.0	0.0	0.6	0.0	0.0
Sweets and candy	22.7	35.8	31.7	19.8	36.7	31.5
- Sugar, honey, jams	11.0	15.5	13.5	7.2	12.0	10.1
- Confectionary	2.6	4.9	4.3	2.9	6.1	5.2
- Chocolate	5.5	9.1	8.0	5.3	9.8	8.3
- Syrups	2.0	3.3	3.3	2.6	5.1	4.7
- Ice cream	1.6	3.0	2.6	1.8	3.7	3.2
Cake and cookies	9.7	12.1	10.5	11.6	15.5	13.1
Non-alcoholic beverages	25.1	29.8	38.3	23.1	23.0	34.4
- Fruit and vegetable juices	7.3	2.7	12.1	9.2	3.5	14.9
- Soft drinks	16.1	25.7	24.0	11.3	18.4	17.4
- Water	0.0	0.0	0.2	1.3	0.0	0.3
- Coffee, tea	1.7	1.4	2.0	1.3	1.1	1.8
Alcoholic beverages	1.6	1.1	1.1	2.4	1.7	1.5
Processed meat	0.6	0.6	0.5	0.4	0.4	0.4
Condiments and sauces	2.8	4.0	3.5	2.3	3.8	3.2
Soups	0.6	1.1	1.0	0.6	1.3	1.1
Miscellaneous	0.4	0.6	0.6	0.8	1.0	0.9

**Table 3:** Adherence to recommendations of added and free sugars (%) in 3817 participants from the Dutch National Food Consumption Survey 2007–2010.

		Added Sugars		Free Sugars	
	N	<10 %TE ^1^ (%)	<20 %TE ^2^ (%)	<5 %TE ^3^ (%)	<10 %TE ^4^ (%)
All	3817	29	83	2	19
Boys, 7–18 years	856	9	70	0	5
Girls, 7–18 years	857	10	75	0	5
Men, 19–69 years	1054	45	91	4	33
Women, 19–69 years	1050	45	92	4	29

^1^ Recommended value by the Nordic Nutrition Recommendations (2012); ^2^ Intake level mentioned by the Dutch health council (2006); ^3^ Recommended value by the SACN (2015) and conditional recommendation by the WHO (2015); ^4^ Recommendation by the WHO (2015).

**Table 4:** Adherence to guidelines of added and free sugars and diet quality as measured with the Dutch Healthy Diet-index (mean (SD)) in 1054 adult men and 1050 adult women from the Dutch National Food Consumption Survey 2007–2010.

	Added Sugars						Free Sugars					
	<10 %TE ^1^	≥10 %TE ^1^	*p*	<20 %TE ^2^	≥20 %TE ^2^	*p*	<5 %TE ^3^	≥5 %TE ^3^	*p*	<10 %TE ^4^	≥10 %TE ^4^	*p*
**Men, 19–69 years**												
N (%)	472 (45)	582 (55)		957 (91)	97 (9)		46 (4)	1008 (96)		347 (33)	707 (67)	
DHD-index, score	59.4 (16.0)	59.7 (12.6)	0.63	59.6 (14.5)	58.4 (11.3)	0.36	59.9 (15.9)	59.5 (14.1)	0.83	58.6 (15.8)	60.0 (13.3)	0.06
Component scores (range 0–10)												
- Physical activity	8.0 (3.6)	8.1 (3.5)	0.89	8.0 (3.6)	8.4 (3.2)	0.30	8.2 (3.7)	8.1 (3.6)	0.81	8.0 (3.6)	8.1 (3.5)	0.58
- Vegetable	6.2 (3.9)	5.6 (3.6)	0.002	6.0 (3.8)	5.1 (3.3)	0.01	6.6 (4.0)	5.9 (3.8)	0.10	6.2 (3.9)	5.8 (3.7)	0.05
- Fruit	4.8 (3.8)	4.5 (3.7)	0.11	4.8 (4.8)	3.2 (4.3)	0.003	5.2 (5.3)	4.6 (4.7)	0.28	4.6 (5.0)	4.7 (4.6)	0.88
- Dietary fiber	6.9 (2.2)	6.0 (1.9)	<0.0001	6.6 (2.1)	4.9 (1.7)	<0.0001	7.2 (2.1)	6.4 (2.1)	0.002	6.9 (2.2)	6.2 (2.0)	<0.0001
- Fish	2.1 (3.8)	1.5 (3.0)	0.0004	1.8 (3.5)	1.1 (2.5)	0.03	1.4 (2.9)	1.8 (3.5)	0.32	2.0 (3.8)	1.6 (3.2)	0.06
- Saturated Fatty Acids	5.0 (4.6)	5.8 (4.0)	0.001	5.3 (4.3)	6.7 (3.8)	0.001	4.9 (5.1)	5.5 (4.3)	0.28	4.9 (4.7)	5.7 (4.1)	0.0003
- Trans Fatty Acids	9.2 (3.3)	9.4 (2.8)	0.25	9.3 (3.1)	9.5 (2.5)	0.52	10.0 (0.0)	9.3 (3.1)	0.05	9.2 (3.4)	9.4 (2.8)	0.15
- Consumption occasions with acidic drinks/foods	9.7 (2.3)	9.5 (2.5)	0.30	9.7 (2.2)	8.7 (3.9)	<0.0001	9.2 (3.6)	9.6 (2.4)	0.12	9.6 (2.5)	9.6 (2.4)	0.95
- Sodium	2.0 (4.3)	1.9 (4.0)	0.60	2.0 (4.2)	2.1 (3.9)	0.82	2.9 (5.1)	1.9 (4.1)	0.06	2.2 (4.5)	1.9 (4.0)	0.24
- Alcohol	5.3 (5.7)	7.4 (4.7)	<0.0001	6.2 (5.4)	8.9 (3.1)	<0.0001	4.4 (5.8)	6.5 (5.3) *	0.001	5.1 (5.6)	7.1 (4.9)	<0.0001
**Women, 19–69 years**												
N (%)	468 (45)	582 (55)		967 (92)	83 (8)		43 (4)	1007 (96)		301 (29)	749 (71)	
DHD-index, score	65.7 (16.1)	64.5 (14.3)	0.11	65.4 (15.2)	60.3 (13.8)	0.001	67.6 (15.3)	64.9 (15.2)	0.12	65.5 (16.3)	64.8 (14.7)	0.40
Component scores (range 0–10)												
- Physical activity	8.8 (3.1)	8.6 (3.0)	0.26	8.7 (3.0)	8.8 (2.9)	0.85	8.3 (4.1)	8.7 (3.0)	0.25	9.0 (2.7)	8.6 (3.1)	0.01
- Vegetable	6.4 (3.9)	5.3 (3.6)	<0.0001	6.0 (3.8)	4.3 (3.1)	<0.0001	7.6 (4.4)	5.8 (3.7)	<0.0001	6.8 (3.8)	5.4 (3.6)	<0.0001
- Fruit	6.0 (4.8)	5.0 (4.5)	<0.0001	5.6 (4.7)	3.5 (4.1)	<0.0001	5.5 (5.2)	5.5 (4.7)	0.96	5.7 (4.9)	5.4 (4.6)	0.28
- Dietary fiber	7.4 (2.3)	6.6 (2.1)	<0.0001	7.1 (2.2)	5.3 (1.7)	<0.0001	8.0 (2.5)	7.0 (2.2)	<0.0001	7.6 (2.3)	6.8 (2.2)	<0.0001
- Fish	2.3 (4.0)	1.6 (3.2)	<0.0001	2.0 (3.7)	1.0 (2.3)	0.005	2.5 (4.8)	1.9 (3.5)	0.14	2.5 (4.1)	1.6 (3.3)	<0.0001
- Saturated Fatty Acids	5.0 (4.8)	5.4 (4.5)	0.14	5.1 (4.7)	6.6 (3.9)	0.001	5.2 (5.4)	5.2 (4.6)	0.98	5.1 (4.8)	5.2 (4.6)	0.53
- Trans Fatty Acids	9.1 (3.7)	8.9 (3.7)	0.52	9.0 (3.6)	8.3 (4.3)	0.04	9.6 (3.7)	9.0 (3.7)	0.16	8.9 (3.9)	9.0 (3.6)	0.73
- Consumption occasions with acidic drinks/foods	9.7 (2.3)	9.8 (1.6)	0.14	9.8 (1.8)	9.3 (2.9)	0.02	9.2 (3.7)	9.8 (1.8)	0.01	9.6 (2.4)	9.8 (1.7)	0.10
- Sodium	4.6 (5.3)	4.7 (5.0)	0.63	4.7 (5.2)	4.7 (4.8)	0.97	5.5 (5.7)	4.6 (5.1)	0.14	4.4 (5.3)	4.8 (5.1)	0.21
- Alcohol	6.4 (5.5)	8.5 (3.8)	<0.0001	7.4 (4.8)	8.5 (3.8)	0.02	6.2 (6.3)	7.5 (4.7)	0.02	5.9 (5.6)	8.2 (4.1)	<0.0001

^1^ Recommended value by the Nordic Nutrition Recommendations (2012); ^2^ Intake level mentioned by the Dutch health council (2006); ^3^ Recommended value by the SACN (2015) and conditional recommendation by the WHO (2015); ^4^ Recommendation by the WHO (2015).

## 4. Discussion

In this representative sample of the Dutch population, total sugar consumption comprised 22 %TE (median: 115 g/day). Added sugars consumption was 12 %TE (median: 64 g/day) and free sugar consumption was 14 %TE (median: 74 g/day). Consumption of total, free, and added sugars was higher in children compared to adults. Foods that contributed most to sugar intake were sugar/honey/jams, chocolate, soft drinks, juices, dairy, and cake and cookies. Adherence to the WHO and SACN recommendations for a free sugar intake <5 and <10 %TE was generally low, especially in children. Overall diet quality was fairly similar between adherent and non-adherent adults to the added and free sugars recommendations.

Direct comparisons between studies are difficult because no uniform definition of added and free sugars exists. The available definitions all state that added sugars are all sugars that are added during manufacturing or preparation [20,23,24,25], not including naturally occurring sugars, such as those in milk and fruit and vegetables [20,24,25]. Free sugars differ from added sugars in their inclusion of naturally occurring sugars in juices, concentrates, honey, and syrups [23]. However, most used definitions of added sugars and free sugars generally show many similarities in their in- and exclusions; therefore, it is not expected to lead to large differences in intakes between studies.

The current consumption of total, free, and added sugars of 22 %TE, 14 %TE, and 12 %TE, respectively, in the Netherlands is comparable to recent intake data from the U.S. and Canada. In 31,305 children and adults of six years and older from the National Health and Nutrition Examination Survey (NHANES) 2003–2010, added sugars provided approximately 14 %TE; contribution of added sugars to energy intake was highest in the 12–17 years age group [26]. In 35,107 Canadians of all ages from the 2004 Canadian Community Health Survey, total sugar intake was estimated at 21 %TE and added sugar intake at 10–14 %TE [27]. Wittekind and Walton have investigated trends in sugars intake reported between 1971 and 2012 in national nutrition surveys from ten European countries, Australia, New Zealand and the United States [28]. In 44 possible comparisons within 13 countries, 7 age- and gender-specific or combined groups, and four categories of sugars, mean population intakes of energy from sugars decreased or remained stable in most comparisons [28]. The current findings are also comparable to intake data from previous surveys in the Netherlands. In the Dutch National Food Consumption Survey 1988, energy intake from total sugars was also 21 %TE [29]. In adults from the DNFCS 1997/1998, mean added sugar consumption was 67 g, which was similar to 66 g consumed by the adults of the present study using data from the DNFCS 2007–2010 [30]. Because previous rounds of the DNFCS used a different dietary assessment method, *i.e.*, a two-day food record, the results cannot be compared directly. However, it suggests that consumption of sugars has not increased or decreased to a great extent in the Netherlands from 1988 to 2010.

To our knowledge, only one other study investigated adherence to the free sugars guideline of the WHO. Among 4140 Australian children and adolescents aged 2–16 years, 1% had an intake <5 %TE and 18.1% an intake <10 %TE [31]. In our study, adherence was lower: only 2% of this representative sample of the Dutch population adhered to the recommendation of the SACN and the conditional recommendation of the WHO of <5 %TE free sugar intake. A larger proportion adhered to the strong recommendation of <10 %TE, but this was still only 5% of the children (7–18 years) and 29%–33% of the adults (19–69 years). As a result, a major public health effort would be needed to increase the adherence to the recommendations. The current study has revealed that within the Netherlands, fruit juices and sugar sweetened beverages, including soft drinks, lemonades, and energy-drinks, contributed most to the intake of free sugar, especially in children. In Australian children, SSBs, cakes, biscuits, pastries, and batter-based products, and sugar and sweet spreads were also the main contributors of added sugar intake [31]. SSBs have been associated with body weight gain and obesity-related diseases [32], which are predominantly mediated by their energy content [33]. However, it has also been shown that persons may not fully compensate their energy intake for the added calories consumed from SSB [8]. Moreover, within NHANES, added sugars intake has already decreased between 1999–2000 and 2007–2008 from 100 g/day (18 %TE) to 77 g/day (15 %TE) [34], which was primarily due to a reduction in sugar sweetened beverage consumption. The authors suggested that this was the result of national efforts as legislations, regulations and campaigns from 1990s and onwards [34]. Therefore, public health efforts in the Netherlands could focus on reducing the consumption of SSB as a strategy to reduce calorie intake. A randomized controlled trial in 641 Dutch children has already shown that a daily consumption of a 250 mL sugar-containing beverage lead to higher weight gain at 18 months compared to consumption of 250 mL sugar-free artificially sweetened beverages [35].

Overall diet quality was not significantly higher in adult women who adhered to the different guidelines for added and free sugars. However, for almost all sugars recommendations, adherent persons scored higher for the components dietary fiber and vegetable intake. On the other hand, those who adhered to the sugar guidelines, tended to score less favorably for the components saturated fatty acid intake and alcohol consumption. This suggests that these adults may have replaced energy intake from sugars by a higher intake of saturated fatty acids and alcohol. The added and free sugars guidelines are expressed as %TE, this inherently indicates that they should be replaced with other energy sources. More research is warranted by which free sugars could best be replaced to improve health, *i.e.*, polysaccharides, proteins, or fatty acids. The current findings are in contrast with most other studies. Louie and Tapsell reviewed the literature on added sugar intake, diet quality, and nutrient intakes. It was observed that a higher intake was associated with poorer diet quality and lower micronutrient intakes [36].

The recommendations of the Health Council of the Netherlands and the NNR with respect to added sugars have been set in relation to micronutrient dilution of the diet. Indeed, a diet high in added sugars has been suggested to dilute micronutrient intake and displace nutrient dense foods in the diet [37]. However, two reviews from 2007 concluded that there is no clear association [38] or only weak evidence [39] between added sugar intake and micronutrient dilution. In addition, a more recent study among Australian children and adults observed that a higher consumption of added sugars, but not total sugars, was associated with nutrient dilution of their diet [40].

A limitation of the present study is that despite our efforts, the added and free sugars content in food products could be under- or overestimated. Food processes, and consequently the food products available in the shops, are repeatedly changing in content and ingredients. For most processed food products, the precise recipes, and, thereby, the added sugar content was unavailable. For these food products, added sugars content was estimated based on information for fulfilment of international Choices Programme criteria, commonly used recipes, information from packaging and comparisons with similar food products with more information available. Furthermore, the Danish food composition table was used for similar Dutch foods which contains information on added sugar content [21]. Added sugar content was assigned to food items by a decision flow-chart categorized by food group, mainly based upon criteria of the international Choices Programme. Because added sugars are not chemically different from natural occurring sugars, most published methods to assign added sugar content include a certain level of subjectivity and are country- and product-specific. To overcome this, Louie *et al.* proposed a 10-step systematic methodology to estimate added sugar content [41]. Although we used a different methodology, which might appear less systematic, we have made similar decisions. We have assigned 0 g added sugars to products with 0 g total sugars (step 1), assigned 0% and 100% added sugars to the same food groups (step 2 and 3), used standard or individual recipes (step 4, 8, and 9), and used analytical data for lactose (step 6), or international data (step 7). However, instead of using the formula to calculate added sugar based on comparison with values from the unsweetened variety (step 5), we have directly subtracted the total amount of sugars minus the amount of natural occurring sugars in an unsweetened variety. This may have led to discrepancies with the Louie *et al.* method.

Strengths of the present study include the use of two non-consecutive 24-h recalls through which some intra-individual variation for estimation of habitual intake could be eliminated using the MSM [22]. Most food composition tables do not include information on the added and free sugar content of foods, leading to use of incomplete food composition table or supply data. Although it is one of the largest challenges of nutritional epidemiology to provide a reliable and correct measure of dietary intake, the use of individual intake data from national surveys is preferred to availability data, since these data do not account for wastage. Hence, the development of a food composition table for added and free sugars was developed and linkage to dietary intake data represents the main strength of the study. Furthermore, the DNFCS 2007–2010 was conducted among a representative sample of the Dutch population.

In conclusion, with a mean intake of 22 %TE total sugars, 14 %TE free sugars, and 12 %TE added sugars, only a small percentage of the population under study adhered to the updated WHO and SACN recommendations. Future studies are warranted on the associations between intake of total and individual sugars intake and cardio-metabolic outcomes and chronic diseases and whether these associations are mediated by their energy content.

## Supplementary Materials

The following are available online at http://www.mdpi.com/2072-6643/8/2/70/s1, Table S1: Decision flow-chart of estimated added sugar content of 2556 foods from the Dutch food composition table 2011 by product groups, largely based upon the criteria of the International Scientific Committee of Choices [20].

## Abbreviations

The following abbreviations are used in this manuscript:

DHD-index: Dutch Healthy Diet-index
DNFCS: Dutch National Food Consumption Survey
MSM: Multiple Source Method
NHANES: National Health and Nutrition Examination Survey
NNR: Nordic Nutrition Recommendations
SACN: Scientific Advisory Committee on Nutrition
SSB: Sugar-sweetened beverages
TE: Total Energy
WHO: World Health Organization
