# Supplementary material for: Total, Free, and Added Sugar Consumption and Adherence to Guidelines: The Dutch National Food Consumption Survey 2007–2010
Source: Nutrients. 2016 Jan 28;8(2):70. doi: 10.3390/nu8020070 (PMC4772034; doi:10.3390/nu8020070)
Supplement: Supplementary file 1 [file nutrients-08-00070-s001.docx]

Supplementary Materials: Total, Free, and Added Sugar Consumption and Adherence to Guidelines: The Dutch National Food Consumption Survey
2007–2010

Diewertje Sluik, Linde van Lee, Anouk I. Engelen and Edith J. M. Feskens

**Table S1.** Decision flow-chart of estimated added sugar content of 2556 foods from the Dutch food composition table 2011 by product groups, largely based upon the criteria of the International Scientific Committee of Choices [20].

| **Product Group** | **Decision** | **N** |
| --- | --- | --- |
| Alcoholic and  non-alcoholic beverages (*n* = 284) | Water, tea, and coffee do not contain added sugars (0%), except when indicated (100%) | 28 |
|  | Fruit juices and fruit concentrates do not contain added sugars (0%); it is assumed that fruit concentrates will be diluted to the original ratio of fruit juices before consumption * | 46 |
|  | Fruit drinks and nectars and fruit drinks with dairy contain 70% added sugar * | 42 |
|  | Energy drinks, flavored waters, and soft drinks contain 100% added sugars; lemonades and syrups contain 75%–85% added sugars * | 108 |
|  | Whey drinks contain 78%–100% added sugars * | 4 |
|  | Light drinks contain 0%–85% added sugars * | 16 |
|  | Alcoholic beverages contain 0% (beer, sherry, wine, cognac, brandy, liquor) to 100% (liqueurs, port, madeira, eggnog) added sugars | 33 |
|  | Rice drink does not contain added sugars (0%). | 1 |
|  | Standard recipe | 6 |
| Breads (*n* = 101) | Plain bread-products contain negligible amount of added sugar (0%). This includes all breads with 0g total sugars, plain crackers, bread and rolls with <6 g total sugars, plain dough, and puff pastry | 70 |
|  | Added sugar content of bread products with fruit is estimated based on subtracting fruit sugars in fruit added to the product from total sugars | 10 |
|  | Added sugar content of bread products with a high total sugar value (>6 g) and non-plain bread products are assumed to be equal to total sugars minus 2 g of natural sugars. This includes all bread products with >6 total sugars, biscuit rusks and toasts, cream crackers, croissants, and sweet breads and rolls | 16 |
|  | Standard recipe | 5 |

**Table S1.** *Cont.*

| **Product Group** | **Decision** | **N** |
| --- | --- | --- |
| Cakes and cookies  (*n* = 210) | Added sugars in cake and cookies are estimated by subtracting the (average) natural sugar value in similar products (which indicate no added sugars) from total sugars | 87 |
|  | Added sugar in snacks is assumed to be equal to the added sugar from similar reference foods expressed as % total sugar, e.g. chocolate coated sweets (85%), brownies (85%), milkshakes (70%), doughnut (95%), waffles (91%), apple-filled pastry (78%), shortbread (95%), sponge cake (97%) | 55 |
|  | Added sugar in snacks with fruit or milk-based fillings are estimated by subtracting natural sugars from the fruits or fillings assuming portion size or assumed to be 90% added sugars | 12 |
|  | Light products do not contain added sugar (0%) | 1 |
|  | Added sugar content in pies is 92%, except fruit pies (38%), apple-nut pies (41%), whipped cream cake (74%), cheese cake (89%) | 3 |
|  | Plain bread and bread products contain negligible amounts of added sugar (0%), including all breads with 0 g total sugars, plain crackers, bread and rolls with <6 g total sugars, plain dough, puff pastry | 3 |
|  | Added sugar content of bread products with a high total sugar value (>6 g) and non-plain bread products are assumed to be equal to total sugars minus 2 g of natural sugars. This includes all bread products with >6 total sugars, biscuit rusks and toasts, cream crackers, croissants, and sweet breads and rolls | 1 |
|  | Standard recipe | 48 |
| Cereals and binding agents (*n* = 104) | Plain flours, flakes, and grains do not contain added sugars (0%) | 53 |
|  | Flour mixes, pancake batters, binding agents (thickeners) do not contain added sugars (0%), except custard powder (100%) | 12 |
|  | Added sugars in breakfast cereals are estimated based on total sugars minus the natural sugars from dried fruits, total sugars minus the natural sugars from grains and cereals (1%) when no other estimation is available, or on total sugars minus natural occurring sugars or total sugars in similar products without added sugars | 36 |
|  | Cornflakes contain 88% added sugars | 1 |
|  | Added sugars in processed cereals and grains (without milk or fruit) is assumed to be equal to total sugar minus 1% natural sugar in grains and cereals (99%) | 2 |
| Cheese (*n* = 64) | Plain cheese and cheese products do not contain added sugars (0%) | 64 |
| Dietetic products  (*n* = 177) | Dietetic products are a negligible source of added sugars in the general population (0%) | 165 |
|  | Glucose powder contains 100% added sugars | 1 |
|  | Cambridge meal replacement contains 100% added sugars | 3 |
|  | Added sugars in Cambridge milkshake is estimated based on total sugars minus natural occurring sugars in plain milk | 1 |
|  | Recipe (website) | 7 |

**Table S1.** *Cont.*

| **Product Group** | **Decision** | **N** |
| --- | --- | --- |
| Eggs (*n* = 7) | Eggs do not contain added sugars (0%) | 7 |
| Fats, oils, savory sauces (*n* = 246) | Fats and oils contain 100% added sugars | 57 |
|  | White sauces contain 100% added sugars, except mayonnaise (57%) and yogurt dressing (97%) | 31 |
|  | Other sauces: curry sauce contains 100% added sugars, BBQ sauce 76%, canned sauces 100%, peanut sauce 100%, gravy 100%, and sweet-sour sauce 100% | 126 |
|  | Ketchup contains 85% added sugars, tomato sauce 80%, and pesto 1.8% | 5 |
|  | Roux and milk- or flour-based sauces do not contain added sugars (0%) | 16 |
|  | Butter (dairy) does not contain added sugars (0%) | 3 |
|  | Fruit sauces (chutney, piccalilli) contain 90% added sugars | 2 |
|  | Water-based sauces without fruit and vegetables contain 100% added sugars | 1 |
|  | Sambal (chili) and shrimp paste do not contain added sugars (0%) | 1 |
|  | Standard recipe | 4 |
| Fish (*n* = 84) | Fresh, cooked, or canned fish do not contain added sugars (0%) | 77 |
|  | Processed fish with sauce, coating, and fillings contains 100% added sugars | 7 |
| Fruit (*n* = 95) | Fresh fruit, boiled, dried or drained fruits, fruits canned in their own juice, fruit concentrates, and fruit and vegetable salads do not contain added sugars (0%) | 72 |
|  | Added sugars in fruits canned in syrup are estimated based on total sugars minus natural occurring sugars in a similar product without syrup | 16 |
|  | Added sugars in fruit-based sauce, puree and compote are assumed to be 40%, with apple sauce as the golden standard used as a reference | 5 |
|  | Added sugars in dairy with fruit are estimated based on total sugars minus sugars from plain milk | 1 |
|  | Standard recipe | 1 |
| Herbs and spices  (*n* = 52) | All herb mixes and stock contain 100% added sugars | 28 |
|  | Dried fruit and vegetables do not contain added sugars (0%) | 15 |
|  | Mustard contains 100% added sugars | 4 |
|  | Sambal and shrimp past do not contain added sugars (0%) | 5 |
| Legumes (*n* = 15) | Plain legumes do not contain added sugars (0%) | 14 |
|  | Beans in tomato sauce contain 1.2% added sugars | 1 |
| Meat and meat products (*n* = 260) | Unprocessed meat and poultry do not contain added sugars (0%) | 173 |
|  | Processed meat (e.g. coated with bread crumbs) contain 100% added sugars | 87 |

**Table S1.** *Cont.*

| **Product Group** | **Decision** | **N** |
| --- | --- | --- |
| Milk and dairy  (*n* = 199) | Plain milk and milk products with no indication of added sugar do not contain added sugars (0%) | 51 |
|  | Added sugars in flavored, enriched, fruited, and sweetened milk products are estimated as total sugars minus sugar from plain milk; milkshakes contain 70% added sugars, condensed milk 42%–44%, and when no information is available added sugars are assumed 100% | 114 |
|  | Milk powder does not contain added sugars (0%); creamer contains 100% added sugars | 4 |
|  | Water-based ice contains 80% to 100% added sugars | 3 |
|  | Light dairy products do not contain added sugars (0%) | 15 |
|  | Iced coffee contains 94% added sugars | 1 |
|  | Plain flours, flakes, and grains do not contain added sugars (0%) | 1 |
|  | Flour mixes, pancake batters, binding agents (thickeners) do not contain added sugars (0%), except custard powder (100%) | 1 |
|  | Standard recipe | 9 |
| Miscellaneous  (*n* = 29) | Sweeteners do not contain added sugars (0%) | 7 |
|  | Unsweetened cacao does not contain added sugars (0%), sweetened cacao contains 100% added sugars | 6 |
|  | Marmite does not contain added sugars (0%) | 1 |
|  | Vinegar does not contain added sugars (0%) | 1 |
|  | Tartex does not contain added sugars (0%) | 1 |
|  | Yeast and salt do not contain added sugars (0%) | 8 |
|  | Fruit does not contain added sugars (0%) | 3 |
|  | Standard recipe | 2 |
| Mixed dishes (*n* = 76) | Mixed dishes contain 100% added sugars; except pasta with bolognaise sauce (45%), pizza crust (18%) | 39 |
|  | Pancakes do not contain added sugars (0%) | 22 |
|  | Dishes without sauce or filling contain 100% added sugars | 3 |
|  | Baby food does not contain added sugars (0%) | 5 |
|  | Water, tea, and coffee do not contain added sugars (0%), except when indicated (100%) | 1 |
|  | Standard recipe | 6 |

**Table S1.** *Cont.*

| **Product Group** | **Decision** | **N** |
| --- | --- | --- |
| Nuts, seeds, snacks  (*n* = 94) | Unroasted, unsweetened, unsalted nuts and seeds do not contain added sugars (0%) | 22 |
|  | Sweetened roasted nuts and seeds contain 100% added sugars | 1 |
|  | Added sugars in snacks are estimated as total sugars minus natural occurring sugars of similar products without  added sugars | 10 |
|  | Savory snacks do not contain added sugars (0%), except shrimp crackers (3%), deep-fried snacks (100%), and kebab (100%) | 51 |
|  | Savory sandwich fillings, e.g. fish salad, contains 100% added sugars | 8 |
|  | Standard recipe | 2 |
| Potatoes (*n* = 56) | Cooked, canned or baked potatoes without sauces or fillings contain a negligible amount of added sugars (0%) | 28 |
|  | Added sugars in processed potatoes, without sauces and fillings, are estimated based on the dish recipes by subtracting natural sugars of the ingredients from total sugars of the dish | 26 |
|  | Plantains do not contain added sugars (0%) | 2 |
| Savory sandwich filling (*n* = 7) | Standard peanut butter does not contain added sugar (0%); added sugars in peanut butter light and peanut butter “3/4” are estimated as total sugars minus sugar content in normal peanut butter | 5 |
| Soups (*n* = 32) | Sandwich spread contains 100% added sugars | 2 |
|  | Stock contains 100% added sugars | 1 |
|  | Cream soups contain 84% added sugars | 6 |
|  | Other soups contain 100% added sugars | 25 |
| Soy and vegetarian products (*n* = 47) | Plain legumes do not contain added sugars (0%) | 2 |
|  | Canned sauces contain 100% added sugars | 3 |
|  | Products with "Valess" contain 100% added sugars | 5 |
|  | Vegetarian products without added ingredients such as cheese do not contain added sugars (0%) | 17 |
|  | Vegetarian products with added cheese are estimated as total sugars minus natural occurring sugars in a similar product without added sugars | 11 |
|  | Soy flour, miso, and soy milk natural do not contain added sugars (0%); soy desserts contain 100% added sugars | 7 |
|  | Standard recipe | 2 |

**Table S1.** *Cont.*

| **Product Group** | **Decision** | **N** |
| --- | --- | --- |
| Sugar, candy, sweet sauces (*n* = 112) | Added sugars in snacks are estimated as total sugars minus natural occurring sugars of products without added sugars, e.g. chocolate, coconut bread, jams, and marmalade | 21 |
|  | Table sugar, honey, syrup, sweet chocolate spread, and sweet fillings contain 100% added sugars; except hazelnut  spread (92%) | 43 |
|  | All candy contains 100% added sugars, except marzipan (95%), and toffee/caramel (75%) | 37 |
|  | Fruit sauces (chutney, piccalilli) contain 90% added sugars | 1 |
|  | Water-based sauces without fruit and vegetables other than ketchup contain 100% added sugars | 2 |
|  | Added sugars in snacks are estimated as total sugars minus natural occurring sugars of similar products without  added sugars | 1 |
|  | Standard recipe | 7 |
| Vegetables (*n* = 205) | Fresh, boiled, baked, canned, pickled, dried, and leaked vegetables without sauces or fillings do not contain added  sugars (0%) | 188 |
|  | Added sugars in pickled vegetables is estimated as total sugars minus total sugars in a similar product without  added sugars | 10 |
|  | Canned tomato products do not contain added sugars (0%) | 4 |
|  | Rhubarb sauces contains 97% added sugars | 2 |
|  | Standard recipe | 1 |

* These products were assumed to contain 100% free sugars; for all other food items added sugars are assumed to be equal to free sugars.
